# Unrolling worms: Genome Editing to Rewrite Roller Phenotypes in *C. elegans*

**DOI:** 10.17912/micropub.biology.002185

**Published:** 2026-06-30

**Authors:** Sohitri Mukherjee, Andrea KH Stavoe

**Affiliations:** 1 Neurobiology and Anatomy, The University of Texas Health Science Center at Houston, Houston, TX, US

## Abstract

The gain-of-function allele *rol-6 ( su1006 )* has been used as a co-injection marker to visually identify transgenic progeny following microinjection in *Caenorhabditis elegans* . The *rol-6 (gf)* allele yields a clear, visual roller phenotype; however, affected worms are twisted along the anterior-posterior axis, obscuring tissues and complicating visual analyses. We deployed CRISPR/Cas9 to “unroll” transgenic worm strains harboring *rol-6 (gf)* . We discovered that our successfully unrolled strains introduced additional nucleotides into the *rol-6 (gf)* loci, rendering the *rol-6 (gf)* copies inactive. Our results indicate that genome engineering can be easily deployed to modify existing transgenic worm strains and could be applied to other gain-of-function co-injection markers.

**Figure 1. Unrolling worms:** (A) Schematic of unrolling technique. (B-C) Quantification of roller phenotype in original and unrolled strains. N ≥ 287 worms across three separate days of quantification for each strain. **** p < 0.0001 by two-tailed Fisher's exact test. (D-G) Representative images of original (D, F) and unrolled worms (E, G). Scale bars, 100 μm. (H-I) Representative micrographs of the nervous system in original (H) and unrolled worms (I). Scale bars, 50 μm. (J) Genotyping gel for *rol-6 ( su1006 )* . Lane 1 is 1kb plus DNA ladder from NEB. (K) Schematic of *rol-6* sequence in front of R71. Wild type *rol-6* nucleotide sequence in black font; corresponding ROL-6 amino acids in bold above codons. Nucleotide changes below wild type *rol-6* sequence: for su1006 in blue, for znyIs15 in magenta, and for znyIs16 in green (79 bp inserted sequence: tgacacactccatatgtctcacagaacaccagtgacctggacacctcacaccggatatggatcgtgaaccggatatgga). Location of nucleotide insertions are depicted by arrows. Associated amino acid changes are depicted above wild type amino acid sequence in corresponding colors.

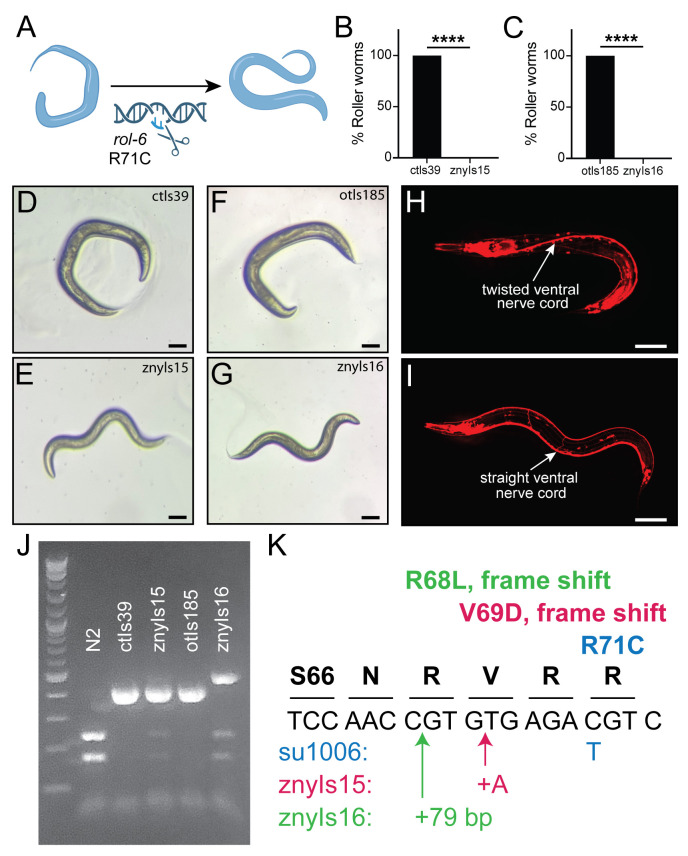



## Description

The ease of generating transgenic worms has been an important advantage in *C. elegans* for decades. Prior to the use of fluorophores as co-injection markers, two popular co-injection strategies took advantage of whole-body phenotypes induced by single point mutations. One strategy used wild-type genes to rescue visible loss-of-function phenotypes. Two examples of this rescuing strategy include *pha-1 (wt)* rescue of *pha-1 (e2123ts)* (Granato et al., 1994) and *unc-119 (wt)* rescue of *unc-119 ( ed3 )* (Maduro and Pilgrim, 1995; Praitis et al., 2001). A second strategy used gain-of-function alleles that induced visible phenotypes injected into wild-type animals. One example of this second strategy is *rol-6 ( su1006 )* to induce a roller phenotype (Kramer et al., 1990; Mello et al., 1991). These strategies either require injecting into mutant animals, which can be challenging, or generating mutant animals, which can affect worm health or the ability to visualize specific phenotypes. Many of these transgenic lines were converted into integrated strains that remain useful today, particularly if the co-injection, whole-animal phenotypes could be removed.

The *rol-6 ( su1006 )* gain-of-function allele, as its name suggests, induces worms to roll (Cox et al., 1980; Park and Horvitz, 1986; Chen et al., 2003; Thein et al., 2003). The *rol-6 ( su1006 )* mutation also induces a developmental delay (Sparling et al., 2023) and lower mating efficiency in males (Evans, 2006). Larval lineage tracing is more difficult in *rol-6 ( su1006 )* larva due to their twisted cuticle (Evans, 2006). In addition, for experiments that involve visualizing the worm nervous system, the ventral nerve cord becomes more difficult to visualize in rol worms than in wild-type animals.

The advent of CRISPR-Cas9 gene editing now makes it possible to engineer single point mutations with surgical precision. CRISPR-Cas9 gene editing has been extensively used in *C. elegans* to edit single loci in animals, with a guide RNA targeting a single locus in the worm genome (Cho et al., 2013; Arribere et al., 2014; Zhao et al., 2014; Paix et al., 2015; Prior et al., 2017; Dokshin et al., 2018). In addition, the SKI LODGE system developed by the Mair lab uses the popular *dpy-10 ( cn64 )* guide RNA to target the endogenous copy of dpy-10 as the co-injection marker and an inserted copy of the dpy-10 guide sequence to edit the SKI LODGE cassette inserted at a safe harbor locus (Silva-García et al., 2019). Thus, efficient guide RNAs can simultaneously target more than one locus in a genome.

We asked whether we could use CRISPR-Cas9 to “un-roll” roller worms made with traditional microinjection techniques. Traditional microinjection techniques generate extrachromosomal arrays that contain many copies of both the experimental plasmid and the co-injection plasmid. Thus, worms with integrated *rol-6 (gf* ) arrays contain an unknown, but likely high, number of *rol-6 (gf)* copies that would need to be reverted to wild-type during “un-rolling.” CRISPR/Cas9 broadly involves two steps: a guide RNA targets the Cas9 enzyme to a specific location to induce a double-strand DNA break and an exogenous repair template provides the sequence to the endogenous repair machinery to generate the desired edit via homologous recombination &nbsp;(Arribere et al., 2014; Zhao et al., 2014; Paix et al., 2015; Prior et al., 2017; Dokshin et al., 2018). We designed a guide RNA targeted to the *rol-6 ( su1006 )* allele and a repair template to replace *rol-6 (gf)* with wild-type *rol-6* . Importantly, in the unlikely event that the endogenous *rol-6* locus were targeted, the repair template should have replaced that edit with wild-type *rol-6* ( Figure 1A ).

We used ribonucleoprotein-complex CRISPR-Cas9 following established methods to introduce the RNA and Cas9 protein via microinjection into adult roller BW1932 (genotype ctIs39 ) worms (Fay et al., 1999). We chose the healthiest line of normally-moving worms and assigned this gene-edited strain a new genotype: znyIs15 (* ctIs39 ). We quantified the number of worms that displayed wild-type versus roller phenotypes in both the ctIs39 and znyIs15 worms. We found that while 100% of ctIs39 worms exhibited roller movement, the unrolled znyIs15 worms all displayed wild-type, sinusoidal movement ( Figure 1B, 1D-E). Finally, we genotyped znyIs15 worms for *rol-6 ( su1006 )* to determine if all *rol-6* array copies were reverted to wild-type. We were surprised to find that our unrolled znyIs15 DNA only displayed a faint WT band ( Figure 1J ). Perplexed, we then sequenced the znyIs15 *rol-6* genotyping PCR product. We discovered that znyIs15 worms harbored a single-nucleotide insertion near the target edit site, inducing V69D and a frame shift, resulting in an early stop codon ( Figure 1K ). The sequencing results suggest that for successfully unrolled worms, the repair machinery ignored the repair template and instead randomly inserted a nucleotide during repair of the majority of array copies of *rol-6* . Further, it appears that this insertion occurred in multiple copies of *rol-6 (gf)* within the array and likely inactivates most of the array copies of *rol-6* . These inactivated copies *rol-6* , combined with a few reverted WT *rol-6* edits, resulted in unrolled worms.

To ensure that this strategy is possible across roller strains developed by different labs, we next unrolled an independent roller strain ( OH6020 ; genotype otIs185 ) (Sarin et al., 2007). Following the same protocol, we were again able to isolate unrolled worms after CRISPR-Cas9 gene editing, assigning this gene-edited strain the genotype znyIs16 (* otIs185 ). Similar to our previous quantifications, we found that the unrolled znyIs16 worms completely lost the roller phenotype of otIs185 worms ( Figure 1C, 1F-G). We also genotyped znyIs16 animals for *rol-6 ( su1006 )* , again finding that unrolled znyIs16 DNA only displayed a faint WT band and a larger-than-expected PCR product ( Figure 1J ). Similar to our znyIs15 sequencing results, we identified a 79-base pair insertion near the edit site in znyIs16 worms. This insertion induced R68L and a frame shift ( Figure 1K ), again generating an early stop codon and likely inactivating most of the array copies of *rol-6* . As with znyIs15, a small fraction of array copies of *rol-6* in znyIs16 do appear to have been reverted to WT. Together, our data indicate that the guide RNA successfully targeted Cas9 to *rol-6 (gf)* loci, but that we chose a suboptimal repair strategy. These results indicate that we can use CRISPR-Cas9 to unroll worms with roller phenotypes induced by the *rol-6 ( su1006 )* gain-of-function allele, but that unrolling worms appears to be more successful by inactivating the array copies of *rol-6 (gf)* than by reverting them to wild type *rol-6* .

We next validated the utility of unrolling worms by visualizing the ventral nerve cord in unrolled worms by generating transgenic lines labeling the nervous system with p rab-3 ::mCherry. Indeed, unrolled worms displayed the typical, wild type untwisted ventral nerve cords that were easier to visualize with confocal microscopy than their roller counterparts ( Figure 1H- I). These data indicate that we can unroll worm strains initially generated with roller co-injection markers instead of laboriously remaking integrated strains with different co-injection markers.

Our data show that CRISPR-Cas9 can be successfully used to edit multiple copies of *rol-6 ( su1006 )* in *C. elegans* , including many copies of *rol-6 (gf)* in multicopy arrays following microinjection and integration. Co-injection markers serve an important purpose in many transgenic strains: they identify the desired transgenic array during crossing and maintenance. Some transgenic arrays are only identifiable via their co-injection markers with a conventional, dissection microscope. Thus, some strains harboring *rol-6 (gf)* co-injection markers may not be easy to work with once unrolled. This is strain-dependent, as we found that we can visualize the GFP signal of ctIs39 /znyIs15 with a fluorescent dissection microscope. One way to address this limitation would be to intentionally insert a cassette into the repair template that could be identified by PCR-based genotyping, similar to the cassette we unintentionally inserted to yield znyIs16. Together, our results identify a new CRISPR-based method to easily revert phenotype-causing *rol-6 (gf)* point mutations from existing transgenic worm strains. This theory may also be applied to other gain-of-function co-injection markers.

## Methods

Genome editing

The rol-6 ( su1006 ) guide RNA (crRNA) and repair template (HDR, ssODN) were designed using Integrated DNA Technologies (IDT) Alt-R™ HDR Design Tool ( https://www.idtdna.com/site/order/designtool/index/HDRDESIGN ).

znyIs15 and znyIs16 were generated using an adapted CRISPR protocol (Ghanta and Mello, 2020). Briefly, the *rol-6 ( su1006 )* crRNA, tracrRNA, and Cas9 protein were mixed and injected into BW1932 and OH6020 adult worms. All CRISPR reagents were obtained from IDT.

Injected worms were singled onto plates and allowed to recover and lay eggs for three days. Then, plates were examined for normal-moving progeny. All normal-moving progeny were singled onto new plates. After another three days, plates were examined for F2 progeny that exhibited normal sinusoidal movement. The healthiest line for each “un-rolling” was selected for subsequent experiments.

Quantifying rol phenotype

We counted L4-adult worms freely moving on plates with OP50 lawns, assaying worms for the roller phenotype versus sinusoidal movement. Approximately 100 worms were quantified per genotype per session. Worms were quantified on three separate sessions on different days.

Transgenesis

Transgenic worms were generated following established techniques. pwAS70 [ rab-3 p::mCh] and pwAS1 [ unc-122 p::gfp] were co-injected at 5ng/uL and 30 ng/uL, respectively, into N2 or VOE643 worms to generate znyEx214 and znyEx220, respectively. znyEx214 was crossed to otIs185 to generate VOE689 .

Microscopy

For white-light images, worms were left on agar with OP50 . Worms were imaged on a dissection microscope at 25x magnification. Images were captured with an iPhone. Images were cropped and rotated in FIJI.

For fluorescent images, worms were mounted on 2% agarose pads and immobilized in 10 mM levamisole (Sigma 31742-250MG). Worms were imaged on a spinning disk confocal microscope (Nikon Ti2 Inverted Confocal with Yokogawa W1 Spinning Disk Package, Nikon Instruments) with a CFI super fluor 40x, 1.3 NA oil immersion objective (Nikon Instruments). Z-stack digital micrographs (step size: 0.5 microns) were acquired with a back-illuminated cCMOS camera (Teledyne Photometrics) using Nikon Elements software (Nikon Instruments).

Statistics

We performed two-tailed Fisher's exact tests with Prism 10 (GraphPad).

## Reagents

*Strain table*

**Table:** 

**Strain**	**Genotype**	**Available From**
N2	Wild type	CGC
BW1932	ctIs39	CGC
OH6020	otIs185	CGC
VOE641	znyIs15 (* ctIs39 )	Stavoe lab
VOE643	znyIs16 (* otIs185 )	Stavoe lab
VOE671	znyEx214 [ rab-3 p::mCh (5 ng/uL), unc-122 p::gfp (30 ng/uL)]	Stavoe lab
VOE689	znyEx214; otIs185	Stavoe lab
VOE690	znyEx220 [ rab-3 p::mCh (5 ng/uL), unc-122 p::gfp (30 ng/uL)]; znyIs16	Stavoe lab

*Plasmids*

**Table:** 

**Plasmid**	**Genotype**	**Description**
pwAS1	unc-122 p::gfp:: unc-54 3' UTR	4kb unc-122 promoter driving codon-optimized gfp, including Ce introns. Available from Addgene.
pwAS70	rab-3 p::mCh:: unc-10 3' UTR	4.4kb rab-3 promoter driving codon-optimized mCherry, including Ce introns. Available from Stavoe lab.

*Genotyping rol-6*

rol-6 ( su1006 ) Fwd: CTG AAA ATT TCC AGA TGA CCC TAA CTA CGG

rol-6 ( su1006 ) Rev: GAA TGG ACC ATC TGG GAA TCC ACC

AatII (New England Biolabs # R3162S) cuts N2 (421 and 31 bp) and *rol-6 ( su1006 )* (242, 179 and 31 bp).

1kb plus DNA ladder (New England Biolabs).

Undigested PCR product was sent for Sanger sequencing with Quintara Biosciences.

*CRISPR-Cas9 reagents*

**Table:** 

**Reagent**	**Available From**	**Catalog number**
Alt-R S.p. Cas9 Nuclease V3, 100ug (Cas9 protein)	IDT	1081058
Alt-R CRISPR-Cas9 tracrRNA, 20 nmol	IDT	203459079
rol-6 ( su1006 ) crRNA	IDT	Sequence: /AlTR1/rUrUrGrUrUrGrArCrArUrCrUrCrArCrArCrGrGrUrGrUrUrUrUrArGrArGrCrUrArUrGrCrU/AlTR2/
rol-6 (wt) HDR (ssODN) (plus strand)	IDT	Sequence: /AlT-R-HDR1/G*T*TGATATGGTTAAACTTGGAGCAGGAACCGCTTCCAACCGTGTGAGACGTCAACAATATGGAGGATATGGAGCCACTGGTGTTCAG*C*C/AlT-R-HDR2/

*Software*

ImageJ (NIH) (Schindelin et al., 2012); Prism 10 (Graphpad). Figure was assembled using Adobe Illustrator.
